# Prevalence of Factor V Leiden mutation in yemeni women with gestational diabetes mellitus and recurrent pregnancy loss: a case-control study

**DOI:** 10.1186/s12920-026-02429-z

**Published:** 2026-07-31

**Authors:** Nabeela A. Al Matari, Abdulkarim M. Al-Obeidi, Ebtesam Al-Zabedi

**Affiliations:** Department of Biochemistry and Molecular Biology, Faculty of Medicine and Health Sciences, Sana’a University, Sana’a, Yemen

**Keywords:** Factor V Leiden, Gestational diabetes mellitus, Recurrent pregnancy loss, Thrombophilia, Miscarriage, Stillbirth, Coagulation, Yemen

## Abstract

**Background:**

Gestational diabetes mellitus (GDM) is one of the most common metabolic disorders during pregnancy and has been associated with recurrent pregnancy loss (RPL) and other obstetric complications. The Factor V Leiden (FVL) is a common inherited thrombophilic mutation. However, data on the prevalence of the FVL mutation among Yemeni women with GDM and a history of RPL remain scarce. Therefore, this case–control study aimed to determine the prevalence of the FVL mutation and examine its association among Yemeni women with GDM and recurrent pregnancy loss compared with healthy pregnant controls.

**Methods:**

This case–control study included 200 Yemeni women with GDM and 200 healthy pregnant controls without a previous history of GDM. Ethical approval was obtained from the Research Ethics Committee of the Faculty of Medicine and Health Sciences, Sana’a University. The GDM group consisted of women with a history of adverse pregnancy outcomes, including spontaneous abortion and/or stillbirth. The FVL (G1691A) mutation was detected by real-time PCR using genomic DNA extracted from peripheral blood leukocytes (white blood cells). Statistical analyses were performed using SPSS version 22.

**Results:**

Allelic frequencies for FVL were 0.93 (G) and 0.07 (A) in the GDM group, compared to 0.98 (G) and 0.02 (A) in controls. The FVL mutation was more common among women with GDM and recurrent pregnancy loss. Clinical findings in the GDM group showed significantly higher PT, APTT, fasting blood glucose, blood pressure, HbA1c, and lipid profile values than healthy controls. Differences in FVL mutation incidence and clinical measures between the GDM and control groups were statistically significant.

**Conclusion:**

Our study found a higher prevalence of the Factor V Leiden (FVL) mutation among Yemeni women with gestational diabetes mellitus (GDM) and a history of recurrent pregnancy loss (RPL) than among healthy pregnant controls. The observed association suggests that the FVL mutation may contribute to the clinical profile of this group. However, further large-scale prospective studies with appropriate comparison groups are needed to validate these findings and to clarify whether FVL screening could have a meaningful role in the clinical management of pregnancies.

## Introduction

GDM is defined as a condition of glucose intolerance with onset or first recognition during pregnancy and hyperglycemia of variable severity [1, 2]. It occurs when the mother’s body is unable to produce enough insulin to overcome her increased resistance associated with pregnancy [3]. Insulin secretion and/or β-cell dysfunction can be disrupted by a range of factors, all of which may contribute to its development one way or another. These include, but are not limited to, advanced maternal age (usually defined as 35 years or more) [4], excess weight before pregnancy, and dietary factors [5]. In Yemen, the prevalence of gestational diabetes mellitus (GDM) has been reported to be around 5.1% in the Dhamar governorate. Identified risk factors include advanced maternal age (≥ 35 years), a family history of diabetes, previous GDM, obesity, and polycystic ovary syndrome [6].

Factor V Leiden (FVL) (G1691A) is a well-characterized genetic mutation. It reduces the normal inactivation of Factor V by activated protein C, thereby predisposing carriers to an increased risk of thrombosis [7]. Research conducted in other countries has demonstrated that FVL is linked to adverse pregnancy outcomes such as recurrent pregnancy loss (RPL [8]), stillbirth, and related complications, most likely due to placental thrombosis and impaired perfusion. Despite these findings, little is known about the contribution of the FVL mutation to pregnancy outcomes among women with GDM in Yemen, leaving an important gap in the regional literature.

### Objectives

Our study aimed to investigate the association between the Factor V Leiden mutation and recurrent pregnancy loss in Yemeni women with gestational diabetes mellitus, as well as its relationship with thrombosis.

## Methods

### Study design

This case–control study was conducted between May 2023 and May 2024; following strict ethical and scientific standards, it was approved by the Research Ethics Committee of the Faculty of Medicine and Health Sciences, Sana’a University. All participants gave informed consent for genetic analysis. A total of 400 pregnant women participated voluntarily, including 200 with normal pregnancies and 200 diagnosed with gestational diabetes mellitus (GDM), aged between 18 and 41 years. Patients with any other disease, such as cardiac disease, liver disease, renal impairment, autoimmune disease, preeclampsia or anticoagulant therapy and diabetes mellitus types 1 and 2 were excluded. All study participants underwent TORCH screening, and none tested positive for any TORCH infection. Participants were recruited from multiple hospitals and maternity centers to ensure a diverse and representative sampling. These included the Departments of Obstetrics and Gynecology at Al-Thawra Hospital, Al-Jomhory Hospital, Al-Sabeen Maternity and Childhood Hospital, and other private hospitals, maternity, and child health centers. Body mass index (BMI) and blood pressure were recorded, and BMI, blood pressure, and laboratory measurements were obtained at the time of participant recruitment. Participants were enrolled during the first, second, and third trimesters of pregnancy. Each participant with GDM was matched with a healthy control recruited during the same gestational period to ensure comparability between the study groups. Body mass index, blood pressure, and laboratory measurements were recorded at the time of recruitment. Fasting blood glucose and the lipid profile were measured on the first day, whereas random blood glucose, complete blood count, prothrombin time, activated partial thromboplastin time, and HbA1c were assessed on the second day.

### Sample size

Based on the global frequency of single-nucleotide polymorphisms (SNPs), the sample size was calculated using Open Epi with a 95% confidence level, the sample size calculation was based on an expected Factor V Leiden allele frequency of approximately 5% in the control population, as reported in previous epidemiological studies of the general population [9] and a statistical power of 80%, as reported in previous studies, an expected odds ratio of 2.0 based on previous literature, The estimated minimum required sample size was approximately 150 participants per group. Therefore, enrolling 200 cases and 200 controls was considered sufficient to ensure adequate statistical power for detecting a significant association.

- *Group 1*: Control (200) Pregnant women with normal pregnancies, without any complications, and in good health.
- *Group 2*: Pregnant women diagnosed with gestational diabetes mellitus (GDM) who had a history of recurrent pregnancy loss.

### Factor V Leiden (G1691A) assay

Genomic DNA was extracted from EDTA-anticoagulated blood samples using a modified salting-out method. Briefly, leukocytes were lysed using sodium dodecyl sulfate (SDS), followed by protein precipitation using a high-salt solution (potassium chloride). After centrifugation, the DNA-containing supernatant was transferred to a new tube, and DNA was precipitated with cold ethanol or isopropanol. The DNA was then washed with ethanol to remove residual impurities and rehydrated in TE buffer (Tris–EDTA), which was used to maintain pH stability and inhibit nuclease activity.

DNA concentration and purity were assessed using a NanoDrop spectrophotometer (JENWAY Genova Nano, UK) by measuring absorbance at 260 nm and 280 nm.

Detection of the Factor V Leiden mutation was performed using Sacace molecular genetic kits based on real-time PCR. Amplification of the target genomic region was carried out using specific primers, and detection was achieved using fluorescent dye-labeled oligonucleotide probes. The accumulation of fluorescent signal was monitored in real time during PCR amplification, allowing detection of the product without opening the reaction tubes.

### Ultrasound and doppler examination

A total of 200 healthy pregnant women underwent ultrasound examination, and all showed viable fetuses. In contrast, among 200 women with gestational diabetes mellitus (GDM), fetal outcomes were demise (Intrauterine fetal death) were observed in approximately six cases carrying the Factor V Leiden GA genotype and in two cases with the AA genotype. Doppler ultrasound examination was performed, revealing evidence of placental thrombosis.

### Sample processing

Following the manufacturer’s protocol for each reagent kit, a Full-automated hematology Sysmex (XN 550) analyzer was used to determine platelet count, and a full-automated coagulation analyzer for PT, PTT (Sysmex CA-500 series). The Mindray BS 240 (Shenzhen Mindray Biomedical Electronics Company Ltd., Shenzheni, China) automated analyzer was used for measuring FBS, RBS, HbA1c, Lipid profile (TC, TG, HDL, LDL), Cobas 411analyser for investigate TORCH infection, and PCR Real-time used by Applied Biosystems 7500 to detect the Factor V Leiden mutation.

### Statistical analysis

Data were analyzed using IBM SPSS (Statistical Package for the Social Sciences) Statistics for Windows (version 22.0; IBM Corp., Armonk, NY, USA). The distribution of continuous variables was first assessed to determine normality. For variables that were not normally distributed, results were expressed as median and interquartile range (IQR). Comparisons between two groups were performed using the Mann–Whitney U test, Hardy–Weinberg equilibrium (HWE) was assessed in the control group; the genotype distribution did not deviate significantly from Hardy–Weinberg equilibrium (HWE), indicating that the population was in HWE, *P* = 0.77 (> 0.05). Logistic regression analysis was used to examine the association between Factor V Leiden genotypes and gestational diabetes mellitus (GDM) as well as pregnancy loss (PRL). A p-value of less than 0.05 was considered statistically significant, and 95% confidence intervals (CI) were reported when appropriate.

## Results

Table 1 shows demographic characteristics of GDM and normal pregnant blood pressure. The findings showed that women with GDM were slightly older than controls, though the women with GDM had significantly higher systolic and diastolic blood pressure as well as higher BMI. The biochemical parameters of normal pregnant and GDM data indicate statistically significant differences between women with normal pregnancies and those diagnosed with GDM. In the GDM group, levels of HbA1c, total cholesterol, triglycerides, LDL-c, and both TyG-related indices were higher compared to the normal group (*p* = 0.001). On the other hand, HDL-c levels were lower among women with GDM. Ratios such as TC/HDL-c and TG/HDL-c were also elevated, pointing to greater disturbances in lipid metabolism and increased insulin resistance.

**Table 1:** Demographic and biochemical parameters between GDM and normal pregnant women

Parameters	GDM	normal pregnant	*p*.value
Age (years)	29 (26–33)	28 (25–32.8)	0.213
< 30	105 (52.5)	116 (58)	
≥ 30	95 (47.5)	84 (42)	
BMI	27.0 (25.3–28.8)	23.5(22.4–24.0)	0.001
Blood pressure systole	110 (106–120)	106 (100–115)	0.001
Blood pressure diastole	70 (70–80)	68 (60–75)	0.001
HbA1 C	6.9 (6.8–7.5)	4.6 (4.2–4.9)	0.001
Cholesterol	139 (125–150)	113 (106–119)	0.001
Triglyceride	139 (122–151)	110 (102–116)	0.001
HDL-c	36 (32–39)	40 (38–42)	0.001
LDL-c	75 (65–86)	52 ( 48–55)	0.001
TYG-index	7.1( 6.3–7.8)	3.8 (3.5–4.1)	0.001
TyG-BMI.index	193 ( 162–220)	88 (79–96)	0.001
TC/HDL-c	3.8 (3.4–4.5)	2.8 (2.7–2.9)	0.001
TG/HDL-c	3.7 (3.2–4.6)	2.8 (2.6–2.9)	0.001

Data and Biochemical parameters were expressed as median and interquartile range (IQR) by the Mann–Whitney U test. *HbA1c* Glycated hemoglobin, *HDL-c* High-Density Lipoprotein cholesterol, *LDL-c* Low-Density Lipoprotein cholesterol, *TyG-index* Triglyceride-Glucose Index, *TyG.BMI.index* Triglyceride-Glucose Body Mass Index, *TC* Total Cholesterol, *TG* Total Triglyceride

Table 2 presents the genotype and allele frequencies of the Factor V Leiden mutation in women with GDM and normal pregnant women who have the Factor V gene mutation.

**Table 2:** Prevalence of Factor V gene mutation and allelic frequencies in GDM and normal pregnant control

Group	Type of mutation	Wild	heterozygous	Odd ratio (95% Cl)	*P*.Value
GDM with RPL (*n* = 200)	Factor V Leiden(G1691A) (Heterozygous and Homozygous mutant) Allelic frequencies G (Wild) 371 (0.93) A (Mutant) 29 (0.07)	173 (0.86)	27 (0.14)	3.75(1.658 − 8.464)	(0.001)
Control without RPL (*n* = 200)	Factor V Leiden(G1691A) (Heterozygous and Homozygous mutant) Allelic frequencies G (Wild) 392 (0.98) A (Mutant) 8 (0.02)	192 (0.96)	8 (0.04)	-	-

Wild (GG) vs. Heterozygous (GA)+ (AA), *GDM* gestational diabetes mellitus, *PRL* recurrent pregnancy loss, *OR* odds ratio, *CL* Confidence interval

Table 3 shows biochemical parameters in GDM according to the mutation. Factor V Leiden has high significant difference between its genotype (GG) vs. GA + AA) and T.C/HDL-c (*p* = 0.015),

**Table 3:** Biochemical parameters in GDM according to the mutation

Parameters	GG	GA	*P*-value
FBS	101 ( 92–180)	103 ( 96–120)	0.112
RBS	172(142– 247)	182 (148–225)	0.072
HbA1c	6.9( 5.6–8.8 )	7.2 ( 6.3–8.6 )	0.054
Cholesterol	139 (100–235)	135( 100–200)	0.776
Triglyceride	139 (83–268)	138 (108–210 )	0.417
HDL-c	36 ( 25–54)	39 ( 30–52 )	0.067
LDL-c	76 (44–152)	71 (48–128 )	0.238
TYG.index	7.1 (4.6–18)	7.0 (5.3–10.8 )	0.987
Tyg.BMI.index	194 (95–482 )	192 (118–302 )	0.589
T.C/HDL-c	3.9 (2.3– 7.8 )	3.5 (2.9–5.1)	0.015
TG/HDL-c	3.8 (2.3–7.8)	3.5 (2.6–5.4 )	0.105

*HbA1c* Glycated hemoglobin, *HDL-c* High-Density Lipoprotein cholesterol, *LDL-c* Low-Density Lipoprotein cholesterol, and TYG-index, Triglyceride-Glucose Index, TyG. *BMI index* Triglyceride-Glucose Body Mass Index, *TC* Total Cholesterol, *TG* Total Triglyceride

Table 4 shows the association of the genotype of Factor V Leiden (G1691A) and coagulation factors. This table shows that Factor V Leiden exhibits a significant difference between its genotypes (GG vs. GA + AA) and PT, Prothrombin Time, APTT, Activated Partial Thromboplastin Time (p-value = 0.001).

**Table 4:** Association of genotype of Factor V Leiden (G1691A) and coagulation factor

Parameters	GG	GA	*P*-value
PT	11.5( 9–20.4)	14.9 (10–17)	0.001
INR	0.88 ( 0.69–1.55)	1.10 (0.76–1.31)	0.001
APTT	26 ( 18–58)	31( 20–35)	0.001

*PT* Prothrombin Time, *APTT* Activated Partial Thromboplastin

Table 5 compares placental thrombosis between women with GDM and normal pregnancies. Placental thrombosis was significantly more frequent in the GDM group compared with controls (*p* = 0.001).

**Table 5:** Comparison of placental thrombosis between GDM and normal pregnancies

Placenta thrombosis	yes	No	*P*-value
GDM	8 (0.04)	192 (96)	0.001
Normal pregnant	-	200 (100)	

*GDM* gestational diabetes melliteus, Placental thrombosis, normal Pregnancies

**Fig. 1:**
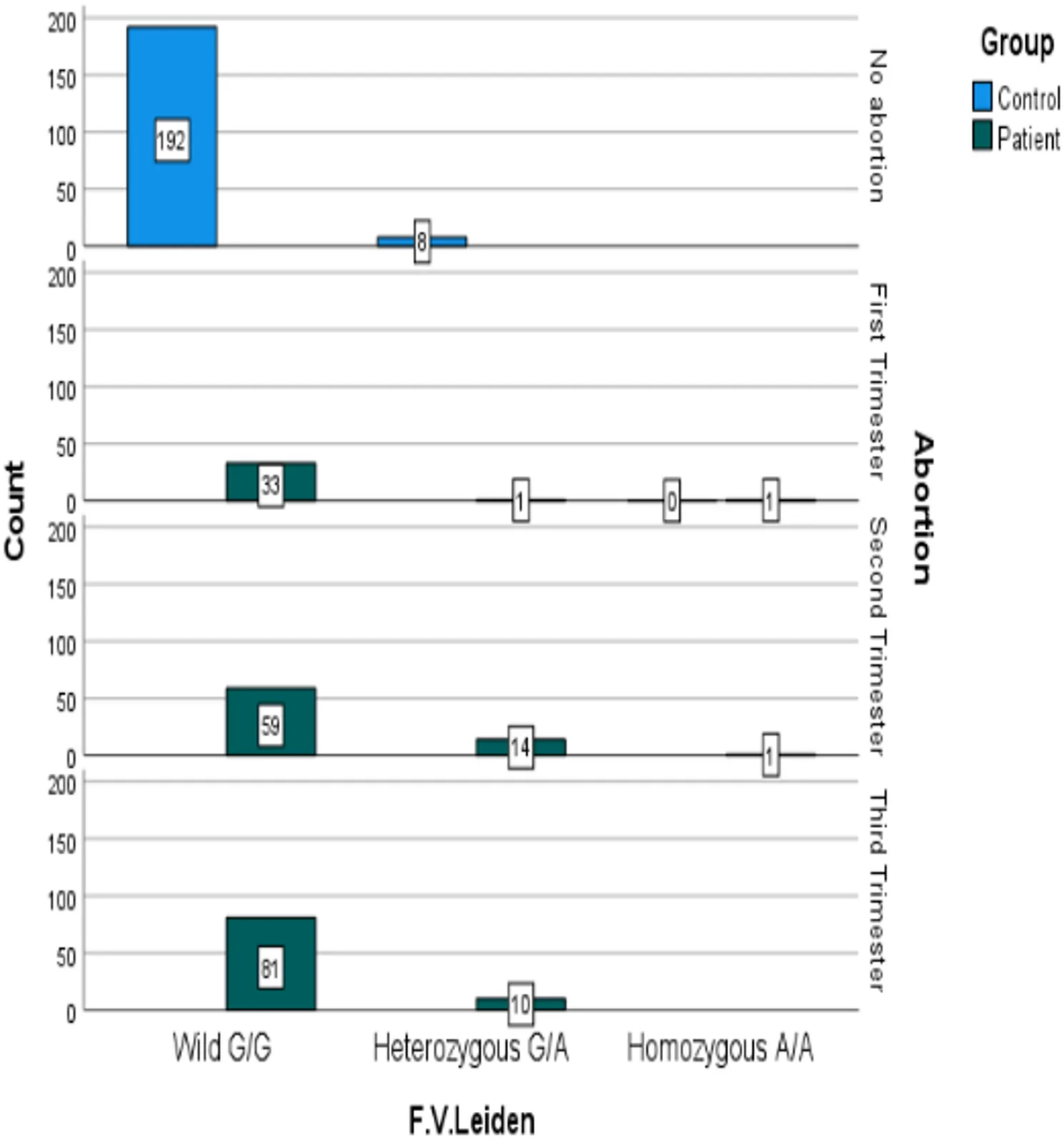
Effect of Factor V Leiden mutation on pregnancy outcomes in gestational diabetes mellitus

Figure 1 shows the effect of F.V.l on trimesters, where GA was in the first trimester, one case, but in the second trimester was 14 cases, and in the third trimester was 10 cases; and AA was one case in the second trimester and one case in the third trimesters.

## Discussion

This study aimed to estimate the association between the Factor V Leiden (FVL) mutation and pregnancy complications in Yemeni women diagnosed with gestational diabetes mellitus (GDM). The results demonstrated that Factor V Leiden represents a key inherited thrombophilic contributor that increases the likelihood of poor pregnancy outcomes. These findings are consistent with previously reported complications including miscarriage [10], intrauterine fetal death [11], and stillbirth [12]. The elevated risk is likely attributed to the prothrombotic state induced by the mutation [13], which promotes the formation of microthrombi in placental vessels, leading to placental insufficiency and restricted fetal growth [14].

In our study, we found that Factor V Leiden was significantly associated with recurrent pregnancy loss (RPL) in women with gestational diabetes mellitus (GDM). Among GDM patients carrying the FV Leiden mutation, those with the heterozygous G/A genotype experienced 25 miscarriages (13%), while those with the homozygous A/A genotype experienced 2 miscarriages (1%). The overall prevalence of Factor V Leiden was 13.5%, with an allele frequency of 7.3%. The mutant A allele (risk allele) of the Factor V Leiden (G1691A) mutation showed a significant association with thrombophilia in GDM, which in turn contributed to miscarriage and stillbirth. These results were consistent with findings reported in Bosnia [15] and in Iran [16].

Logistic regression analysis demonstrated a statistically significant association between the Factor V Leiden mutation and gestational diabetes mellitus. Women carrying the mutation had approximately 3.7-fold higher odds of developing GDM than non-carriers (OR = 3.746, 95% CI: 1.658–8.464, *p* < 0.05). Women who carry the FVL mutation are more likely to develop GDM than those without it, This suggests that inherited thrombophilia may influence glucose metabolism during pregnancy. These results are consistent with the thrombophilic mutations, which worsen vascular and endothelial dysfunction, and they in turn, contribute to placental infarctions due to insufficient blood to the placenta, which leads to miscarriage or stillbirth, as found by a study in Romania [17], and also a corresponding study was done in Iran [18], In contrast, The The detection of the Factor V Leiden mutation in 4% of the control group likely reflects its background prevalence in the general population rather than a disease-specific association. Previous studies have shown that the mutation can also be identified in apparently healthy individuals, although its prevalence varies considerably among different ethnic groups, being highest in European populations and substantially lower in Asian and African populations. Prevalence varies from 10% to 15% in southern Sweden and Greece to 2%-3% in Italy and Spain [13]. The factor V Leiden variant is extremely rare in Asian, African, and indigenous Australian populations [13]. Increased prevalence of the factor V Leiden variant was reported in individuals with cerebral venous thrombosis in a meta-analysis including 1,822 affected individuals and 7,795 controls (odds ratio [OR] 2.70, 95% CI 2.16–3.38) [19].

Overall, this finding may suggest that carrying the mutation alone is not enough to directly cause disease. Instead, it seems more likely that it contributes to disease risk when combined with other genetic or environmental factors. This could explain why some individuals in the control group carry the mutation but remain asymptomatic. Taken together, these results reinforce the concept that thrombotic and pregnancy-related complications are influenced by multiple interacting factors. Therefore, the clinical significance of this mutation should be interpreted with caution, particularly when it is identified in individuals without evident symptoms.

Prothrombin time (PT) is used to evaluate the extrinsic and common coagulation pathways, including factors I, II, V, VII, and X. Activated partial thromboplastin time (APTT) assesses the intrinsic and common pathways involving factors I, II, V, VIII, IX, XI, and XII. Together, these tests provide complementary information on coagulation status and help identify hemostatic abnormalities [20, 21]. On the other hand, prolonged prothrombin time (PT) was found to have a protective effect, suggesting that women with higher PT values may be less susceptible to thrombosis-related pregnancy complications. highlighted the importance of maintaining balanced coagulation parameters to ensure proper placental perfusion and healthy fetal development.

Our study indicated that the GA genotype showed a notably longer PT of 14.9 seconds, especially since none of these patients were on blood thinners. This likely stems from a ‘consumption coagulopathy’ effect; this study was in parallel with studies carried out in London [22]. In this mechanism, their bodies are constantly forming tiny, subclinical micro-clots; they are ‘burning through’ their available supply of clotting factors. Consequently, when the blood is tested in the lab, it actually takes longer to clot simply because the necessary proteins have already been used up elsewhere.

Under normal conditions, activated protein C (APC) inhibits both factor Va and factor VIIIa, thereby slowing down thrombin (factor IIa) generation and preventing hypercoagulation. However, in the presence of the Factor V Leiden mutation (a guanine-to-adenine substitution at nucleotide 1691 in the *F5* gene, resulting in the Arg506Gln variant), factor Va becomes resistant to inactivation by APC [14].

As a result, Factor Va remains active for a longer period, leading to increased activation of Factor X to Xa. Factor Xa, in turn, converts more prothrombin (factor II) into thrombin. The excess thrombin production continuously activates factors V, VII, and VIII, creating a state of chronic hypercoagulability (microthrombosis), Over time, a form of excessive consumption of coagulation factors (consumption coagulopathy) develops because the body persistently forms tiny microclots that consume factors II, VII, and X. This process can lead to a prolonged prothrombin time (PT) in laboratory testing [22]. This mechanism is known as “consumption coagulopathy secondary to chronic microthrombosis”, in which the prolonged PT results not from impaired synthesis of clotting factors, but from their continuous consumption in microthrombotic events caused by resistance to activated protein C [23],Our findings on allele frequencies and the association between Factor V Leiden (FVL) and recurrent pregnancy loss (RPL) are consistent with previously reported regional data. In a study conducted among Sudanese women, Babker et al. identified FVL (G1691A), prothrombin (G20210A), and MTHFR (C677T) mutations in women with RPL, further supporting the role of inherited thrombophilic variants in adverse pregnancy outcomes [24], also In Sudan, additional studies have also demonstrated significant associations between RPL and other inherited variants, including angiotensin-converting enzyme (ACE I/D) polymorphism, human platelet antigen (HPA-1 and HPA-3), factor XIII mutations, prothrombin gene variants, and MTHFR polymorphisms [25, 26].

The present findings further support that placental abruption represents a classic example of this pathophysiological mechanism, particularly in cases of concealed abruption or when it is complicated by stillbirth [23]. Although some researchers attribute the coagulopathy associated with placental abruption primarily to a consumption mechanism, the process appears to be more complex. In fact, women who develop retroplacental clots often experience a more rapid onset and severe form of disseminated intravascular coagulation (DIC), compared to those with postpartum hemorrhage (PPH), despite having a lower overall blood loss. This observation suggests that placental abruption induces a mixed coagulopathy, combining both consumption coagulopathy and the massive release of thromboplastin (tissue factor) from the damaged placental tissue. Overall, this study adds valuable regional data and strengthens the global understanding that the Factor V Leiden mutation plays a pivotal role in the development of complications among women with gestational diabetes mellitus.

Additionally, BMI, systolic blood pressure (SBP), and diastolic blood pressure (DBP) were significantly higher in women with GDM compared to non-GDM pregnant women (*p* = 0.001). General obesity is a known predictor of GDM, but it remains unclear whether specific types of obesity—such as central or visceral adiposity—may pose differential risks [27]. The association between random blood sugar (RBS) level was significantly higher in individuals with the GA genotype compared to those with the GG genotype, indicating that the A allele of the Factor V Leiden mutation may contribute to recurrent pregnancy loss (RPL) in patients with gestational diabetes mellitus (GDM) and is associated with increased glucose levels this result agree with study was done in Caucasians where the prevalence of factor V leiden is 3–15% this result is consistent with Padda et al. [14].

Furthermore, lipid-related indices, including TyG index, TyG-BMI, TG/HDL-c, and TC/HDL-c, were significantly elevated in women with GDM [28]. A case-control study was conducted in China. Of 1586 pregnant women, 741 were diagnosed with GDM, which found that TG/HDL-c and TC/HDL-c were higher in GDM than in normal pregnancies. Compared to healthy pregnant women, those with gestational diabetes mellitus (GDM) tend to show significantly higher triglyceride (TG) levels throughout pregnancy. The GDM group also displays increased total cholesterol (TC), low-density lipoprotein (LDL), and TG/HDL ratios, along with reduced high-density lipoprotein (HDL) levels. Therefore, both TG levels and the TG/HDL ratio may serve as reliable indicators and potential risk factors for identifying GDM, consistent with the study in Serbia [29]. Also, significantly higher levels of HbA1C, total cholesterol, LDL-c & triglycerides, and low HDL were found in patients with GDM as compared to normal pregnant. This finding comparable to study done by Khan, R.et al. [30]; where the relationship between HbA1C and lipid profile occurs when insulin function decreases, resulting in the increased; lipase-sensitivity hormone (HSL) that will cause lipolysis, so that it will release fatty acids and glycerol, which will be carried to the blood circulation, consequently, free fatty acids increase so that if in excess amount, they will be taken to the liver for fat metabolism.

Overall, this study confirms a significant association between the FVL mutation and increased risk of thrombophilia in women with GDM (OR = 3.75, *p* = 0.001). Placental thrombosis, particularly in homozygous carriers, may contribute to miscarriage through impaired placental perfusion. This finding aligns with a previous study conducted in Romania by Petronela Vicoveanu et al. [31]. The presence of blood clots in the placenta can disrupt the flow of oxygen and nutrients to the fetus, potentially leading to miscarriage due to placental abruption [18]. These findings are particularly valuable in the Yemeni context, where genetic screening for thrombophilia is not yet routine [32] and research on the genetic aspects of GDM remains limited. Identifying FVL as a possible risk factor highlights the importance of integrating genetic testing with metabolic assessment in prenatal care [33]. Early detection of women carrying the mutation could allow for closer follow-up [34], preventive management, and potentially better pregnancy outcomes [34].

This study has several limitations that should be considered when interpreting the findings. First, it was conducted in a single region of northern Yemen with a relatively small sample size, which may limit the generalizability of the results and reduce the statistical power of some analyses, particularly for the homozygous AA genotype due to the small number of carriers. Second, maternal age, body mass index (BMI), and blood pressure differed between the study groups and were not adjusted for in a multivariable logistic regression model; therefore, residual confounding cannot be excluded. Third, the case-control design does not allow causal inferences regarding the relationship between the Factor V Leiden mutation and adverse pregnancy outcomes. Fourth, only the Factor V Leiden mutation was investigated, while other inherited thrombophilic variants(APC resistance, D-dimer, fibrinogen, ) and potential environmental or lifestyle-related factors, including nutritional status, were not evaluated. Fifth, the study did not include separate GDM-only and RPL-only comparison groups, limiting the ability to distinguish the independent contribution of each condition to the observed associations. Finally, challenging local conditions may have affected data collection and laboratory procedures. Future multicenter studies with larger sample sizes, broader genetic panels, adjustment for potential confounding factors, and four comparison groups (GDM + RPL, GDM-only, RPL-only, and healthy controls) are warranted to better define the independent contribution of each condition and validate the present findings.

## Conclusion

This study provides new regional evidence supporting the association between the Factor V Leiden mutation, coagulation abnormalities, and adverse pregnancy outcomes in women with gestational diabetes mellitus (GDM). The findings reinforce that GDM is a multifactorial condition in which genetic susceptibility and hemostatic disturbances may interact to influence maternal and fetal outcomes. In settings such as Yemen, where genetic screening for inherited thrombophilia is not routinely performed during prenatal care, these findings suggest that the Factor V Leiden mutation may serve as a potential genetic marker in Yemeni women. Although routine screening cannot be recommended based on the present findings alone, integrating genetic and coagulation assessments in carefully selected patients may improve stratification and facilitate closer clinical monitoring. Further large-scale, multicenter prospective studies are required to confirm these findings and determine their clinical utility before routine implementation in prenatal care.

## Data Availability

The datasets used and/or analyzed during the current study are available from the corresponding author on reasonable request.

## Abbreviations

APC: Activated protein C
APTT: Activated partial thromboplastin time
GDM: Gestational diabetes mellitus
FVL: Factor V Leiden
PT: Prothrombin Time
HbA1c: Glycated hemoglobin
HDL-c: High-Density Lipoprotein cholesterol
LDL-c: Low-Density Lipoprotein cholesterol
and TYG. index: Triglyceride-Glucose Index, TyG.BMI.index, Triglyceride-Glucose
BMI: Body mass index
TC: Total cholesterol
TG: Triglyceride
